# Insights into the Stability and Lipid Oxidation of Water-in-Oil High Internal Phase Emulsions: Roles of the Concentration of the Emulsifier, Aqueous Phase, and NaCl

**DOI:** 10.3390/foods14091606

**Published:** 2025-05-01

**Authors:** Jiao Wei, Jingwen Shang, Yanxiang Gao, Fang Yuan, Like Mao

**Affiliations:** 1 Key Laboratory of Healthy Beverages, China National Light Industry, College of Food Science and Nutritional Engineering, China Agricultural University, Beijing 100083, China; weijiao1205@126.com (J.W.); shang1219jingwen@126.com (J.S.); yxgao@cau.edu.cn (Y.G.); yuanfang0220@cau.edu.cn (F.Y.); 2 CAU Sichuan Chengdu Advanced Agricultural Industrial Institute, Chengdu 611430, China

**Keywords:** W/O emulsions, high internal phase, stability, lipid oxidation, interface

## Abstract

Water-in-Oil high internal phase emulsions (W/O HIPEs) have great potential in developing novel healthy food products. However, the high content of the aqueous phase poses great risks in physical stability and lipid oxidation. This study aimed to understand the relationship between physical stability and lipid oxidation of W/O HIPEs, focusing on the roles of emulsifiers, aqueous phase volume, and NaCl concentration. The findings revealed that increasing the polyglycerol polyricinoleate (PGPR) concentration (10 wt%) significantly enhanced physical stability and slowed lipid oxidation at various temperatures. W/O emulsions with varying aqueous phase volumes (30–80%) maintained good physical stability; however, a higher aqueous phase volume significantly accelerated lipid oxidation. Furthermore, the inclusion of NaCl (10–300 mM) improved the physical stability of W/O HIPEs but also accelerated lipid oxidation. Notably, W/O HIPEs with 50 mM NaCl showed both optimal physical and oxidative stability. Additionally, based on the fitting equation of the primary oxidation products, it was predicted that the oxidation reaction of the W/O emulsion followed a zero-order oxidation kinetics model. By altering the structure of the emulsion system, the physical stability and lipid oxidation stability of the emulsion could be regulated, thereby extending the storage time of food products. Overall, these findings emphasized the critical role of interfacial properties in lipid oxidation, providing new insights for optimizing food formulations to enhance long-term stability.

## 1. Introduction

Lipid oxidation is a challenging issue in foods high in oils and fats, particularly those containing unsaturated fatty acids. Lipid oxidation is the reaction between the unsaturated bonds in lipid molecules and oxygen molecules, forming peroxides and other oxidation products. Oxidation processes during food processing and storage may produce rancid odors and harmful compounds, which detrimentally impact both flavor and nutritional value. Lipid oxidation generally occurs via three main pathways: enzymatic oxidation (triggered by enzymes), photo-oxidation (induced by light-activated sensitizers), and auto-oxidation [1]. The first two conditions can usually be well controlled through heat treatment and packaging. To slow down auto-oxidation in foods, approaches such as adding antioxidants (green tea extract) [2], metal chelators (EDTA) [3], controlling processing conditions, and modifying food formulations are commonly used.

In Oil-in-Water (O/W) emulsions, lipid oxidation is predominantly triggered at the oil–water interface, which is affected by interfacial properties, droplet size, pro-oxidants, antioxidants, and others [4]. Research has indicated that lipids in O/W emulsions are more prone to oxidation than in bulk oils, largely because the oil–water interface enhances the interaction between unsaturated fatty acids (UFAs) and aqueous phase pro-oxidants. Conversely, in Water-in-Oil (W/O) emulsions, lipid oxidation involves the oil–water and oil-air interfaces, with the continuous oil phase exposed directly to air. This adds complexity to the understanding of lipid oxidation in W/O emulsions [5]. W/O high internal phase emulsions (W/O HIPEs), with the aqueous phase volumes exceeding 74%, present significant challenges in controlling lipid oxidation due to their distinctive structures. In these emulsions, water droplets are highly packed to form polygonal shapes, contributing to high viscoelasticity and self-supporting properties. These distinct characteristics necessitate a different stabilization approach compared to conventional W/O emulsions [6]. Polyglycerol polyricinoleate (PGPR) is widely regarded as an excellent lipophilic semi-synthetic emulsifier that can be used alone or in combination with other emulsifiers to form W/O or W/O/W emulsions. It is commonly used in the production of low-fat spreads and the delivery of active ingredients. Previous studies have demonstrated that increasing the PGPR concentrations could enhance the anti-coalescence and sedimentation stability of W/O emulsions by reducing droplet size and increasing the viscosity of the emulsion [7]. Additionally, using 2 wt% PGPR could maintain complete aqueous phase stability in artificial butter after freeze–thaw cycles [8]. However, for W/O HIPEs, a large amount of PGPR was typically required for stabilization, and high concentrations of PGPR could lead to off-flavors in food products. To avoid the excessive use of PGPR, other stabilization mechanisms were applied to assist the roles of PGPR. One such method involves gelling either the aqueous or oil phase, which has been shown to be effective in stabilizing W/O HIPEs [9]. In Lee’s study, W/O HIPEs with ultra-high aqueous phase content were formulated by incorporating carrageenan into the aqueous phase and monoglycerides into the oil phase [10]. We have also demonstrated that adding NaCl to the aqueous phase also enhanced the stability of W/O HIPEs in previous studies [11]. Despite numerous studies focusing on physical stability, a comprehensive investigation into lipid oxidation within W/O HIPEs has yet to be conducted.

Lipid oxidation at the oil–air interface was greatly controlled by extraneous factors (e.g., temperature, oxygen, and light). Oxygen was crucial for hydroperoxide formation, a key step in lipid oxidation, while light exposure accelerated oxidation by producing singlet oxygen through photosensitization. Elevated temperatures further expedited both lipid oxidation and emulsion degradation [12]. Lipid oxidation occurred at the oil–water interface and was controlled by the properties of the interface and aqueous phase [13,14]. The interface served as a binding site for interactions between lipids, oxygen, and pro-oxidants, with smaller droplets and thicker interfacial membranes enhancing oxidation by increasing interaction sites [15]. Emulsifiers could modulate oxidative stability by influencing the behavior of pro-oxidant metals, hydroperoxides, antioxidants, and metal chelators. For instance, phosphorylated perilla protein isolate (LZPI)-protocatechuic acid (CSPA) complexes were found to slow lipid oxidation in O/W HIPEs by forming protective interfacial films [16]. Transition metals, due to their hydrophilic nature, diffused readily into the oil–water interface, catalyzing lipid oxidation by producing free radicals (e.g., hydroxyl and peroxyl radicals) [17]. In addition, the lipid oxidation process might also be affected by the composition of the aqueous phase. NaCl could enhance the physical stability but played complicated roles in the lipid oxidation of W/O emulsions. At lower concentrations, NaCl could act synergistically with proteins to reduce lipid oxidation by sequestering transition metals in O/W emulsions [18]. However, at higher concentrations (>500 mM), NaCl might promote oxidation, as observed in whey protein isolate-stabilized emulsions [19]. In some cases, NaCl had minimal impact on lipid oxidation [20]. In fact, in many previous studies, high concentrations of transition metals were present in emulsions, as well as proteins, both of which could mask the roles of the salt. Therefore, more profound study was required to elucidate the roles of salt in lipid oxidation in W/O emulsions.

In this study, W/O HIPEs with an aqueous phase fraction of 80% were prepared, and the mechanism of lipid oxidation was explored. To achieve the goals, we investigated the effects of (i) emulsifier concentration, (ii) aqueous phase volume fraction, and (iii) NaCl concentration on the physical stability and lipid oxidation of W/O emulsions. Lipid oxidation was analyzed through peroxide value (POV) and thiobarbituric acid reactants (TBARS). Additionally, based on the Arrhenius Model, the oxidation rate of W/O emulsions at different temperatures was predicted. We aimed to provide insights into the complex dynamics of lipid oxidation in W/O high internal phase emulsions.

## 2. Materials and Methods

### 2.1. Materials

Polyglycerol polyricinoleate (PGPR) was obtained from Yousuo Chemical Technology Co., Ltd. (Shandong, China). Canola oil was derived from Yihai Kerry Arawana Holdings Co., Ltd. (Shanghai, China). Potassium thiocyanate, trichloroacetic acid, 2-thiobarbituric acid, and 1,1,3,3-tetraethoxypropane were derived from Shanghai Macklin Biochemical Technology Co., Ltd. (Shanghai, China). The remaining reagents were of analytical grade.

### 2.2. Fabrication of W/O Emulsions

W/O emulsions were prepared according to the previous method [21]. Initially, 4, 6, 8, and 10 wt% PGPR were blended with canola oil and stirred at 45 °C for 10 min, serving as the continuous phase. Meanwhile, the aqueous phases were generated using 10, 50, 100, 200, and 300 mM NaCl added to deionized water. 30, 50, and 80% (*v*/*v*) of the dispersed phase was gradually added to the continuous phase while applying continuous shear using Ultra-Turrax T18 digital shears (IKA, Staufen, Germany). To minimize variations in oxygen content during experiments, we standardized the mixing to 2 min at 5000 rpm and shearing to 5 min at 7000 rpm to prepare W/O emulsions. Tinfoil was used to prevent exposure to light during the whole process. Table 1 shows the composition of different W/O emulsions. The W/O HIPEs (80% aqueous phase and 200 mM NaCl) with 4, 6, 8, and 10 wt% of PGPR were labeled as P-4, P-6, P-8, and P-10, respectively. The W/O HIPEs (80% aqueous phase and 8 wt% PGPR) with 10, 50, 100, 200, and 300 mM NaCl were labeled as Na-10, Na-50, Na-100, Na-200, Na-300. The W/O emulsions (8 wt% PGPR and 200 mM NaCl) with 30, 50, 80% (*v*/*v*) dispersed phase were labeled as D-30, D-50, D-80.

### 2.3. Physical Stability and Physical Instability Index (PII)

The stability of W/O HIPEs was assessed using the LUMiSizer stability analyzer (L.U.M., Berlin, Germany), employing accelerated destabilization through centrifugation [22]. In brief, the samples underwent centrifugation at 4000 rpm for 3600 s, with a 10 s pause during the procedure. The relationship between transmission, time, and position was evaluated, while the emulsion physical instability index (PII) was also recorded.

### 2.4. Measurement of Lipid Oxidation Parameters

The lipid oxidation of the samples was evaluated using the Schall Oven Test (SOT). All samples were stored in glass vials and subjected to oxidation through stored at 4, 37, and 55 °C. The primary oxidation products formed at 0, 3, 6, 9, and 12 days at 4, 37, and 55 °C were analyzed to determine the kinetic pattern of lipid oxidation. Additionally, the secondary oxidation products formed at 0, 3, 6, 9, 12, and 15 days at 55 °C were also determined.

#### 2.4.1. Peroxide Value (POV)

The Peroxide Value (POV) method was used to quantify lipid hydroperoxides, the primary products of oxidation [23]. A total of 5–80 mg of the samples were added to 5 mL of the chloroform/methanol mixture (7:3, *v*/*v*), 25 µL of Potassium thiocyanate solution and 25 µL of FeCl_2_ solution were given. The absorbance values at 500 nm were recorded with a UV-Vis spectrophotometer (Shimadzu, Model UV 1800, Kyoto, Japan) and quantitative calculations were carried out with the standard curve of Fe^3+^. The results were expressed as milliequivalent oxygen per kilogram of oil (meq O_2_/kg of oil), and were calculated using Equation (1):

(1) $$\mathrm{POV}(\mathrm{meq}/\mathrm{kg})=\frac{A}{55.84\;\times \;2\;\times \;m}\;\times \;\frac{1000}{1000}$$

where *A* was the Fe^3+^ mass (µg) in the sample solution obtained from the standard curve and *m* was the mass (g) of samples.

#### 2.4.2. Thiobarbituric Acid Reactants (TBARS)

Malondialdehyde (MDA) was the main secondary oxidation product and was determined by the thiobarbituric acid reactants (TBARS) method [18]. A total of 15–200 mg of the emulsions were taken to glass tubes with screw stoppers. Afterward, 2 mL of TBA reagent was poured. The tubes were subjected to a boiling water bath for 30 min followed by centrifugation. To eliminate air bubbles, they were then sonicated. The absorbance values of the supernatant were determined at 532 nm. The concentrations of TBARS in the emulsion were calculated from the standard curve of 1,1,3,3-tetraethoxypropane.

### 2.5. Oxidation Kinetic Model

In the accelerated oxidation experiment, the primary oxidation products were fitted to determine the oxidation kinetics, order of oxidation, and oxidation rate (*k*) of the W/O emulsion. The Arrhenius equation quantitatively described the connection between temperature (*T*) and reaction rate (*k*) [2]. The logarithmic form of the Arrhenius Equation (2) was as follows:

(2) $$\ln\left(k\right)=\frac{-E_{a}}{RT}+lnA$$

where *k* was the reaction rate constant, *E_a_* was the activation energy (kJ/mol), *R* was the gas constant (8.314 J/(mol·K)), *T* was the absolute temperature (K), and *A* was the pre-exponential factor. Activation energy (*E_a_*) refers to the minimum energy required for the reaction to occur, while the pre-exponential factor (*A*) represents the collision frequency and effectiveness of the molecules.

### 2.6. Statistical Analysis

All experiments were carried out in triplicate. Experimental data are presented as the mean ± standard deviation (SD) and were analyzed using Origin 2019 and SPSS 20.0 software. Significant differences were determined by analysis of variance (ANOVA) and further identified using Duncan’s multiple range test (*p* < 0.05).

## 3. Results and Discussion

### 3.1. Physical Stability and Storage Stability of W/O HIPEs

**Figure 1:**
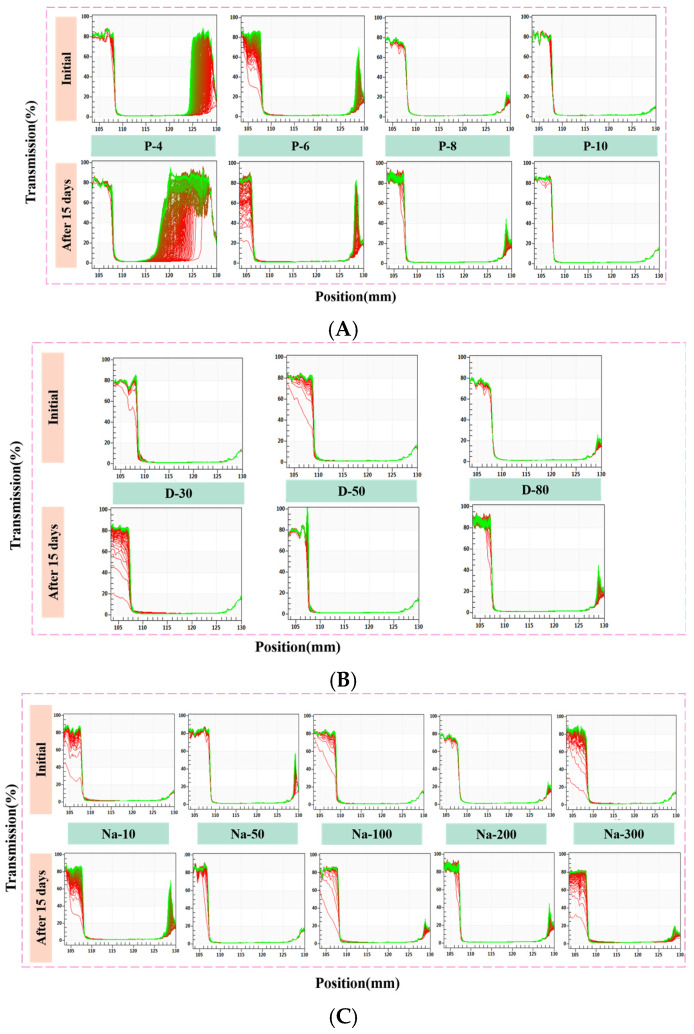
Physical stability of W/O HIPEs via LUMiSizer. (**A**) PGPR concentration; (**B**) aqueous phase volume; (**C**) NaCl concentration. P-(4–10) refers to W/O HIPEs containing 80% (*v*/*v*) aqueous phase and 200 mM NaCl, with 4, 6, 8, or 10 wt% PGPR. D-(30–80) refers to W/O emulsions containing 8 wt% PGPR and 200 mM NaCl, with 30, 50, or 80% (*v*/*v*) aqueous phase. Na-(10–300) refers to W/O HIPEs containing 8 wt% PGPR and 80% (*v*/*v*) aqueous phase, with 10, 50, 100, 200, or 300 mM NaCl. The red curve represents the initial scan spectrum, while the green curve represents the final scan spectrum.

Physical stability, at the initial state and after storage for 15 days, was evaluated by accelerated centrifugation tests with the LUMiSizer. Figure 1A illustrates how the transmission of W/O HIPEs with varying concentrations of PGPR (P-(4–10)) changed over time. Initially, P-4 and P-6 showed a significant increase in transmission at the top and bottom of the test tubes when centrifuged, indicating phase separation within the emulsions. After 15 days, P-4 showed a widening change in transmission at the bottom, evidencing intensified sedimentation of the aqueous phase. This indicated that PGPR at the concentration lower than 4% was insufficient for long-term stability. In contrast, P-6 maintained good stability during storage, while P-8 and P-10 exhibited lower transmission values throughout the test, highlighting their good physical stability. Notably, the above results were obtained in an accelerated test, and only P-4 showed significant phase precipitation after 15 days under quiescent conditions. It was thus concluded that at least 8 wt% PGPR was required to preserve good physical stability of W/O HIPEs. The ultra-high aqueous phase volume (80%) increases their susceptibility to droplet aggregation and phase separation [10]. PGPR can quickly adsorb to the oil–water interface, preventing droplets from aggregating and inhibiting phase separation [24].

For W/O emulsions with 30, 50, and 80% (*v*/*v*) aqueous phase (D-(30–80)), all the samples exhibited quite low transmission values initially, indicating good physical stability (Figure 1B). After 15 days, the transmission at the top of D-30 was increased significantly, demonstrating some oil release. In contrast, transmission values of D-50 and D-80 remained constant throughout the storage test. At 30% aqueous phase volume, droplets were larger and maintained a regular spherical shape, which was more flowable and prone to aggregation. Increasing the aqueous phase volume will result in smaller droplets. This transition may result in droplets with irregular polygonal shapes, lower fluidity, and increased viscosity [25], contributing to higher stability.

The effect of NaCl on the stability of W/O HIPEs was also evaluated. Figure 1C illustrates that W/O HIPEs with varying concentrations of NaCl (Na-(10–300)) show only minor changes in transmission at the top, suggesting relatively good stability. However, the transmission of Na-10 was much higher, probably because some oil was released. After 15 days, the transmission of Na-10 at the top and bottom rose significantly, highlighting significant phase separation during storage. In contrast, the transmission of Na-50, Na-100, and Na-200 was not significantly changed during the whole test, showing good stability against increased NaCl concentration. The findings were in line with those in the literature [26]. This indicated a positive correlation between emulsion stability and NaCl concentration. The presentation of NaCl into the aqueous phase can moderate the Ostwald ripening phenomenon [27]. However, a higher concentration of NaCl will lead to some oil release in the upper layer, indicating decreased emulsion stability. This phenomenon may stem from higher concentrations of NaCl decreasing the ability of the emulsifier to lower interfacial tension.

**Figure 2:**
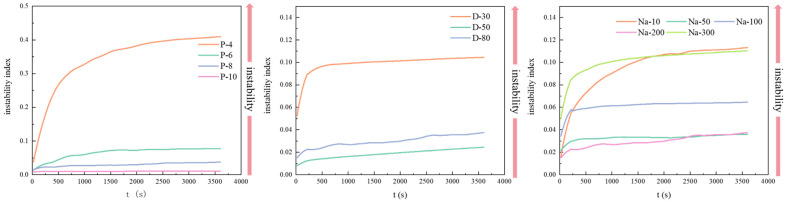
The physical instability index for W/O emulsions stabilized by different concentrations of PGPR (P-4, 6, 8, 10), NaCl (Na-10, 50, 100, 200, 300), and aqueous phase volume after 15 days (D-30, 50, 80).

The physical instability index (PII) is used to predict the physical stability of the sample during long-term storage. It represents the degree of separation of the sample during the centrifugation process, with a value range from 0 (stable) to 1 (unstable) [28]. When the PII is <0.1, the sample is considered to have excellent stability, with almost no phase separation, making it suitable for long-term storage. Figure 2 shows the physical instability index for W/O emulsions after 15 days. The PII of P-4 after 15 days was greater than 0.4, indicating significant phase separation, making it unsuitable for long-term storage. As the concentration of PGPR increased, the instability index was decreased to below 0.1 (P-(6–10)), demonstrating that the emulsion could maintain good stability during long-term storage. W/O emulsions at different aqueous phase volume fractions exhibited a relatively lower PII, but the PII of D-30 was close to 0.1, which might indicate slight oil phase release during storage (Figure 1). In the presence of different concentrations of NaCl, the PII of the W/O HIPEs was generally lower, indicating that the addition of NaCl contributed to maintaining good physical stability during storage. Additionally, it should be noted that slight phase separation may occur in Na-10 and Na-300 during storage.

### 3.2. Lipid Oxidation

#### 3.2.1. Effect of Exogenous Factors on Lipid Oxidation of W/O Emulsions

**Figure 3:**
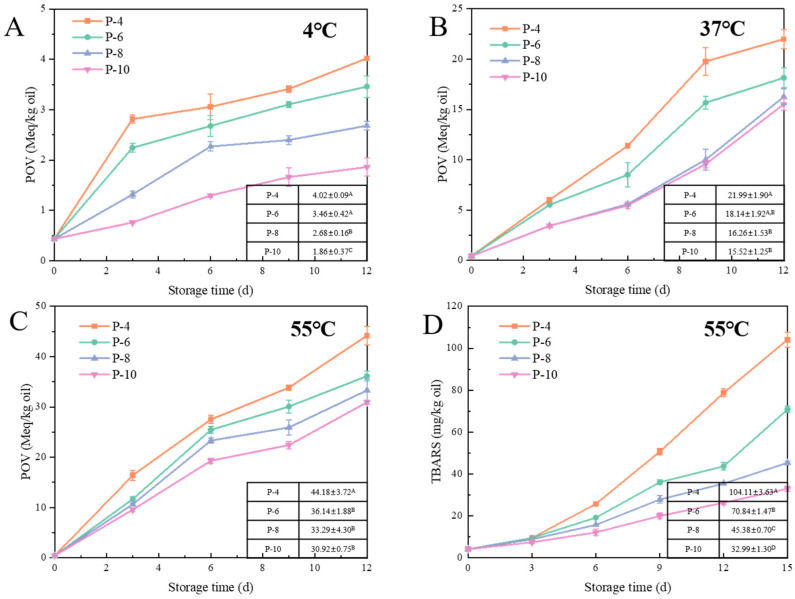
Formation of lipid oxidation markers: POV at 4 °C (**A**), 37 °C (**B**), 55 °C (**C**), and TBARS values at 55 °C (**D**), in W/O HIPEs with different concentrations of PGPR. The POVs on day 12 and TBARS values on day 15 of W/O HIPEs are presented in the table of the figure. P-(4–10) refers to W/O HIPEs containing 80% (*v*/*v*) aqueous phase and 200 mM NaCl, with 4, 6, 8, or 10 wt% PGPR (different superscript letters (A, B, C …) in the figure represent significant differences (*p* < 0.05)).

**Figure 4:**
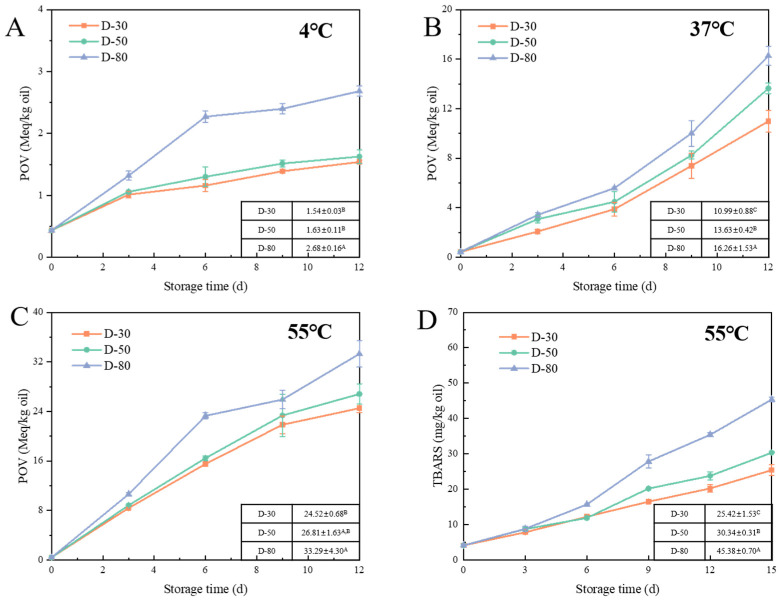
Formation of lipid oxidation markers, POV at 4 °C (**A**), 37 °C (**B**), 55 °C (**C**) and TBARS values at 55 °C (**D**), in W/O emulsions with different aqueous phase volumes. D-(30–80) refers to W/O emulsions containing 8 wt% PGPR and 200 mM NaCl, with 30, 50, or 80% (*v*/*v*) aqueous phase. The POVs on day 12 and TBARS values on day 15 of W/O emulsions are presented in the table in the figure (different superscript letters (A, B, C …) in the figure represent significant differences (*p* < 0.05)).

**Figure 5:**
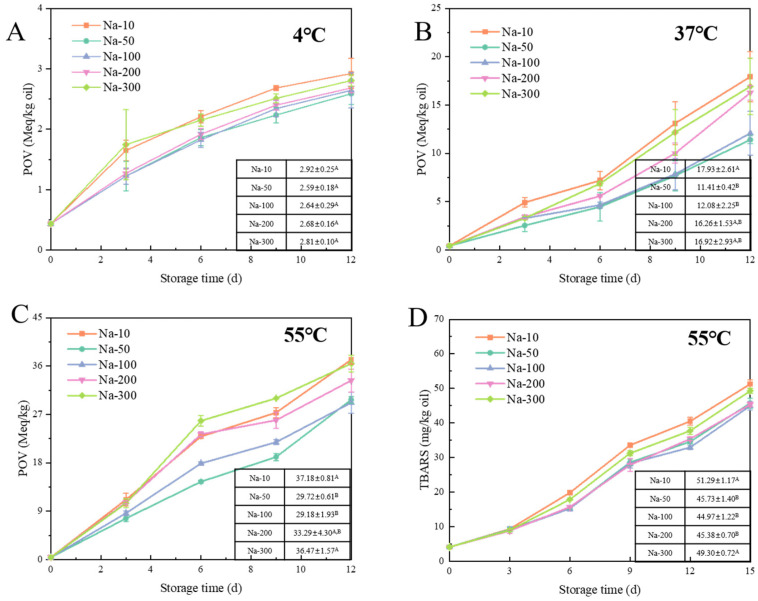
Formation of lipid oxidation markers, POV at 4 °C (**A**), 37 °C (**B**), 55 °C (**C**) and TBARS values at 55 °C (**D**), in W/O HIPEs with different concentrations of NaCl. The POVs on day 12 and TBARS values on day 15 of W/O HIPEs are presented in the table of the figure. Na-(10–300) refers to W/O HIPEs containing 8 wt% PGPR and 80% (*v*/*v*) aqueous phase, with 10, 50, 100, 200, or 300 mM NaCl. (Different superscript letters (A, B) in the figure represent significant differences (*p* < 0.05)).

Lipid oxidation was assessed by quantifying lipid hydroperoxides and TBARS, which indicated the production of primary and secondary oxidation products. Key exogenous factors influencing lipid oxidation include storage time, temperature, light, and oxygen. For all emulsions, both primary and secondary oxidation products were increased during storage. With the increase in storage time, the oxidation rate was typically increased. This was because the unsaturated fatty acids in the lipid were exposed to oxygen for a longer period, thereby increasing the opportunity for oxidation reactions. To examine the effect of temperature, changes in lipid hydroperoxide were analyzed for the samples stored at 4, 37, and 55 °C. As illustrated in Figure 3, Figure 4 and Figure 5, elevated temperatures significantly accelerated the process of lipid oxidation [29]. After 12 days at 4 °C, the peroxide value (POV) of the W/O HIPEs was below 4 Meq/kg oil. In contrast, the POV of the samples stored at 37 °C was about five times higher; at 55 °C, it increased to about ten times higher. The corresponding reaction rate constant (*k*) at individual temperatures was calculated (Table 2, Table 3 and Table 4). The oxidation rate constant was increased exponentially with the rise in temperature. When samples were stored at 37 °C, the larger *k* value was 3.39 (P-4) and the smaller *k* value was 2.05 (D-30). When samples were kept at 4 °C, the larger *k* value was 0.25 (P-4), and 0.08 (D-30). Higher temperatures speed up lipid oxidation by increasing the movement of molecules, making it easier for oxygen to react with the fats. This will lead to faster breakdown of lipids and the formation of oxidation products, causing the food to spoil more quickly. To minimize the impact of oxygen and light on lipid oxidation, we standardized the sample preparation time and used tinfoil to shield the samples from light throughout the experiments.

#### 3.2.2. Effect of Emulsifier on Lipid Oxidation of W/O HIPEs

In W/O emulsions, emulsifiers play crucial roles in controlling lipid oxidation by modulating the interfacial properties. Figure 3 illustrates the relationship between PGPR concentration (P-(4–10)) and the formation of primary and secondary oxidation products, with corresponding reaction rate constants (*k*) presented in Table 2. Lipid oxidation in W/O HIPEs was slowed to varying extents with higher concentrations of PGPR. After 12 days at different temperatures, compared to P-4, the POVs of the other samples were significantly lower (*p* < 0.05). The oxidation rate constant (*k*) was increased with the rise in PGPR concentration. The *k* of P-4 at 55 °C was 1.17 times higher than that of P-6, 1.29 times higher than that of P-8, and 1.42 times higher than that of P-10. The generation of secondary oxidation products was not only influenced by the emulsion compositions but was also affected by hydroperoxides. Consequently, there was no significant variation in TBARS values among all the samples at the initial stage of oxidation. The addition of PGPR at higher concentration significantly decreased the generation of secondary oxidation products at the later stage of oxidation. After 15 days, the TBARS value for P-4 was 104.11 ± 3.63 mg/kg oil, while it was only 32.99 ± 1.30 mg/kg oil for P-10. It could thus be seen that an increase in emulsifier concentration would retard the process of lipid oxidation in W/O HIPEs.

PGPR, as a non-ionic emulsifier, is generally believed to function by reducing interfacial tension, thereby affecting the distribution of pro-oxidants or antioxidants at the interface. In fact, emulsions of different physical stability may result in different oxidation stability of the lipids. In systems with poor physical stability, droplets may coalesce, cream, or precipitate, accelerating the process of oxidation reactions spreading from one droplet to another and increasing contact with metal ions (iron ions) [30]. Therefore, the increase in PGPR concentration also delays the oxidation reaction. Moreover, the inhibition of lipid oxidation may be linked to the formation of anti-clusters in the oil phase due to excessive PGPR [31]. Hydrophobic emulsifiers are present not only at the oil–water interface but also form reverse micelles in the oil phase when in excess. In this study, when the PGPR concentration reached 8 wt%, the emulsion still exhibited good stability after 15 days (Figure 1). Therefore, we believed that 8 wt% PGPR was sufficient for the formation of highly stable W/O HIPEs. As more PGPR was added, excess PGPR would form reverse micelles [32]. Previous research demonstrated that PGPR reverse micelles could enhance the solubility of lipid hydroperoxides, transferring them from the oil–water interface to the continuous phase, thereby slowing lipid oxidation in W/O emulsions [33].

#### 3.2.3. Effect of Aqueous Phase Content on Lipid Oxidation of W/O HIPEs

Lipid oxidation in W/O emulsions is significantly affected by the volume of the aqueous phase. As illustrated in Figure 4, a higher volume of the aqueous phase was correlated with higher levels of primary oxidation products. After 12 days at different temperatures, the POV of D-80 was significantly higher than that of D-30 and D-50 (*p* < 0.05). In contrast, there was no significant difference in the primary oxidation reactions of D-30 and D-50 after 12 days. By fitting the primary oxidation reaction curves, it was found that the oxidation rate constants (*k*) of D-80 at different temperatures were 2.15 times (4 °C), 1.45 times (37 °C), and 1.31 times (55 °C) that of D-30, respectively (Table 3). At lower temperatures, the effect of the aqueous phase volume fraction on the lipid oxidation rate (*k*) is more significant. The secondary oxidation products remained insignificantly different in the early stages of oxidation. After 9 days, the TBARS value of D-80 was significantly higher than that of the other samples. After 15 days, the TBARS values turned out to be 25.41 ± 1.53 mg/kg oil for D-30, 30.34 ± 0.31 mg/kg oil for D-50, and 45.37 ± 0.69 mg/kg oil for D-80. These results indicated that a greater fraction of the aqueous phase would accelerate lipid oxidation of W/O emulsions.

There was very limited research concerning the effects of aqueous phase volume on lipid oxidation in W/O emulsions. Studies on O/W systems showed that an increase in dispersed phase volume slowed lipid oxidation [34]. It has been suggested that a higher oil phase fraction reduced the concentration of water-soluble pro-oxidants and soluble oxygen, thereby inhibiting free radical production per droplet and slowing lipid oxidation [35]. In W/O emulsions, the larger number of droplets in samples will result in closer packing and thus higher oil–water interfacial area, which can provide more sites for lipids to interact with pro-oxidants. Additionally, the content of metal ions in the aqueous phase may also rise. Although the water used in this study was deionized, metal equipment (such as IKA shears) used in emulsification could introduce metal ion contamination, enhancing oxidative effects [36]. Moreover, the proximity of droplets facilitates the propagation of oxidation reactions through direct exchanges of reactive species, such as hydroperoxides and free radicals. As the aqueous phase volume increased, the number of droplets was increased, leading to a greater likelihood of reaction transfer between neighboring droplets [37].

#### 3.2.4. Effect of NaCl on Lipid Oxidation of W/O HIPEs

Figure 5 also shows the dynamics of lipid oxidation of W/O HIPEs with varying concentrations of NaCl (Na-(10–300)). The concentration of the primary oxidation products showed an initial decrease followed by an increase along with the increase in NaCl concentration. Notably, the oxidation rate constants (*k*) of Na-10 were significantly higher than Na-50 (Table 4). This observation, supported by LUMiSizer results during storage (Figure 1), suggested that the increased oxidation rate in Na-10 might be linked to droplet migration and emulsion instability [38]. After 12 days at 37 °C and 55 °C, the POV of Na-300 was significantly higher than that of Na-50 and Na-100 (*p* < 0.05). However, at 4 °C, there was no significant difference in the POV after 12 days. As the storage time increased further, the oxidation rates of W/O HIPEs tended to converge. The effect of NaCl concentration was also less significant for the generation of secondary oxidation products. After 15 days, the TBARS values were 51.28 ± 1.16 mg/kg oil (Na-10), 45.73 ± 1.40 mg/kg oil (Na-50), 44.96 ± 1.22 mg/kg oil (Na-100), 45.37 ± 0.69 mg/kg oil (Na-200), and 49.29 ± 0.72 mg/kg oil (Na-300), respectively. In addition to Na-10, an increase in NaCl concentration accelerated lipid oxidation in W/O emulsions.

Literature studies showed contradictory results on the effects of NaCl. Some studies indicated that salt-protein interactions would retardate lipid oxidation, but these effects varied de pending on the specific samples involved [16]. Other studies detected a pro-oxidant effect of NaCl in O/W emulsions, likely due to the increased solubility and catalytic activity of iron ions facilitated by chloride ions. In addition, monovalent salts (e.g., NaCl or KCl) may accelerate lipid oxidation by altering the arrangement of surface-active molecules, thereby enhancing pro-oxidant interaction with the oil phase [39]. In practical applications, such as margarine, NaCl was linked to increased lipid oxidation [40]. This underscored the complex role of NaCl in oxidative stability, highlighting the need for careful consideration of NaCl concentration in formulations to manage oxidative processes effectively.

### 3.3. Oxidation Kinetic Model of W/O Emulsion

The oxidative rancidity of lipids can severely affect food quality. However, food is a complex system, and the oxidation kinetics are influenced by various factors. In the Section 3.2, the POVs from accelerated oxidation experiments of W/O emulsions were fitted, and the reaction order of oxidation kinetics was determined through regression coefficients. The relationship between POVs and storage time for zero-order kinetics should be satisfied by *P* = *kt* + *P*_0_, while first-order oxidation kinetics should be satisfied by *P* = *P*_0_ *exp* (*kt*) [41]. In the fitting of this experiment, the POVs increased linearly with storage time, which conformed to the zero-order oxidation kinetics (R^2^ = 0.76–0.98). And the reaction rate constants (*k*) were obtained (Table 2-4). On the other hand, the first-order oxidation kinetics fitting showed a smaller R^2^ (0.60–0.94), and some data could not be fitted, so the data were not presented. In studies of edible oils, such as olive oil, soybean oil, and tengkawang butter, the variation in POVs also conformed to the zero-order kinetics equation [42,43]. However, the oxidation of flaxseed oil nano-emulsion followed first-order kinetics, which might be related to various factors such as storage time, temperature, and others [41]. Subsequently, the relationship between the reaction rate constant and temperature was explored using the Arrhenius equation.

Table 5 shows the regression curve between *ln*(*k*) and 1/*T*, with a regression coefficient (R^2^) greater than 0.98. By calculation, the activation energy (*E_a_*) and pre-exponential factor (*A*) for different samples were obtained. When the *E_a_* value is large, it means that the *k* is more sensitive to temperature, and the reaction requires higher energy to proceed [44]. At lower temperatures, the larger *A* value of the sample, the higher its *k*. The *E_a_* values of all samples were relatively low, indicating that the oxidation reaction could occur at lower energy (temperatures) [2]. As the PGPR concentration increased, the W/O HIPEs exhibited a larger *E_a_* value. This suggested that the increase in PGPR concentration could increase the sensitivity of lipid oxidation in W/O HIPEs to temperature, and the rate of lipid oxidation could be delayed by controlling the temperature. Compared to D-80, the W/O emulsions (D-30 and D-50) exhibited larger *Ea* values, indicating higher sensitivity to temperature and the need for higher energy input for the reaction. However, the *A* values of D-30 and D-50 also increased significantly, suggesting a higher frequency of effective molecular collisions. This might be related to the fact that the W/O emulsions with a higher oil phase content were more fluid in nature. The *Ea* values of W/O HIPEs with different NaCl concentrations showed minimal variation. However, the *A* values of Na-10 and Na-300 were increased significantly, indicating an increase in the active sites for molecular collisions. This might be due to the lower physical stability of Na-10 and the pro-oxidative effect of 300 mM NaCl. However, lipid oxidation involves multiple steps, including initiation and propagation. The Arrhenius equation assumes that the activation energy remains constant throughout the reaction. As a complex multiphase reaction system, lipid oxidation in W/O emulsions is influenced by factors such as multiphase oxygen diffusion and free radical chain reactions. Using the Arrhenius equation to predict the relationship between reaction rate and temperature is limited in practical applications.

The W/O emulsions prepared in this experiment included both liquid and semi-solid forms. They were widely used in food, serving as fat substitutes in margarine and also enabling the targeted delivery of hydrophilic bioactive substances [45]. Lipid oxidation is a key factor that affects food quality. Based on the above analysis, it could be concluded that by altering the composition and structure of the W/O emulsions, the progression of lipid oxidation at different temperatures can be controlled. In addition to lipid oxidation, the quality of food is also influenced by various essential factors such as flavor, texture, and mouthfeel [46]. Improving food quality from multiple aspects is one of the tasks that need to be addressed in the future.

## 4. Conclusions

This study investigated the impact of PGPR concentration, aqueous phase volume, and NaCl concentration on the physical stability and lipid oxidation of W/O emulsions. The results demonstrated that increasing the concentration of PGPR significantly improved the physical stability of the W/O HIPEs. Among the various formulations, those with a higher concentration (10 wt%) of PGPR exhibited enhanced oxidative stability. The emulsions with different aqueous phase volume fractions showed good physical stability. Conversely, a higher aqueous phase volume (80%) accelerated lipid oxidation. Notably, the presence of NaCl improved the emulsions’ physical stability, but its higher concentrations also contributed to a faster lipid oxidation rate. The W/O HIPEs containing 50 mM NaCl showed the best balance between physical and oxidative stability. Based on the fitting equation of the primary oxidation products, it was predicted that the oxidation reaction of the W/O emulsion followed a zero-order oxidation kinetics model. By altering the structure of the emulsion system, the physical stability and lipid oxidation stability of the emulsion could be regulated, thereby extending the storage time of food products. The insights gained from this study can inform the design of food products with favorable W/O emulsion structures.

## Figures and Tables

**Table 1:** The composition of different W/O emulsions.

Sample	PGPR Concentration (wt%)	Aqueous Phase Volume Fraction (*v*/*v*)	NaCl Concentration (mM)
P-4	4	80%	200
P-6	6		
P-8	8		
P-10	10		
D-30	8	30%	200
D-50		50%	
D-80		80%	
Na-10	8	80%	10
Na-50			50
Na-100			100
Na-200			200
Na-300			300

**Table 2:** The POVs at different temperatures were fitted in W/O HIPEs with different concentrations of PGPR. P-(4–10) refers to W/O HIPEs containing 80% (*v*/*v*) aqueous phase and 200 mM NaCl, with 4, 6, 8, or 10 wt% PGPR.

Sample	4 °C	37 °C	55 °C
P-4	y = 0.2589x + 1.1937 (R^2^ = 0.74)	y = 1.8962x + 0.5439 (R^2^ = 0.97)	y = 3.4974x + 3.3416 (R^2^ = 0.97)
P-6	y = 0.2303x + 1.0020 (R^2^ = 0.81)	y = 1.5192x + 0.5383 (R^2^ = 0.97)	y = 2.9935x + 2.7933 (R^2^ = 0.95)
P-8	y = 0.1859x + 0.7051 (R^2^ = 0.87)	y = 1.2748x−0.5049 (R^2^ = 0.94)	y = 2.7001x + 2.5147 (R^2^ = 0.94)
P-10	y = 0.1252x + 0.4521 (R^2^ = 0.97)	y = 1.2102x−0.3769 (R^2^ = 0.95)	y = 2.4603x + 1.7693 (R^2^ = 0.97)

**Table 3:** The POVs at different temperatures were fitted in W/O emulsions with different aqueous phase volumes. D-(30–80) refers to W/O emulsions containing 8 wt% PGPR and 200 mM NaCl, with 30, 50, or 80% (*v*/*v*) aqueous phase.

Sample	4 °C	37 °C	55 °C
D-30	y = 0.0863x + 0.5895 (R^2^ = 0.89)	y = 0.8799x − 0.3260 (R^2^ = 0.96)	y = 2.0542x + 1.8182 (R^2^ = 0.97)
D-50	y = 0.0947x + 0.6184 (R^2^ = 0.87)	y = 1.0520x − 0.3385 (R^2^ = 0.93)	y = 2.2422x + 1.7255 (R^2^ = 0.97)
D-80	y = 0.1859x + 0.7051 (R^2^ = 0.87)	y = 1.2748x − 0.5049 (R^2^ = 0.94)	y = 2.7001x + 2.5147 (R^2^ = 0.94)

**Table 4:** The POVs at different temperatures were fitted in W/O HIPEs with different concentrations of NaCl. Na-(10–300) refers to W/O HIPEs containing 8 wt% PGPR and 80% (*v*/*v*) aqueous phase, with 10, 50, 100, 200, or 300 mM NaCl.

Sample	4 °C	37 °C	55 °C
Na-10	y = 0.2003x + 0.7764 (R^2^ = 0.89)	y = 1.4393x + 0.0797 (R^2^ = 0.98)	y = 2.9886x + 1.8645 (R^2^ = 0.98)
Na-50	y = 0.1773x + 0.6046 (R^2^ = 0.96)	y = 0.9034x − 0.1134 (R^2^ = 0.97)	y = 2.3337x + 0.2789 (R^2^ = 0.98)
Na-100	y = 0.1846x + 0.5856 (R^2^ = 0.97)	y = 0.9292x + 0.0897 (R^2^ = 0.96)	y = 2.3567x + 0.0897 (R^2^ = 0.98)
Na-200	y = 0.1859x + 0.7051 (R^2^ = 0.87)	y = 1.2748x − 0.5049 (R^2^ = 0.94)	y = 2.7001x + 2.5147 (R^2^ = 0.94)
Na-300	y = 0.1837x + 1.1456 (R^2^ = 0.85)	y = 1.3952x − 0.4352 (R^2^ = 0.98)	y = 3.0530x + 2.3207 (R^2^ = 0.94)

**Table 5:** Relevant parameters predicted for the W/O emulsion based on the Arrhenius model. P-(4–10) refers to W/O HIPEs containing 80% (*v*/*v*) aqueous phase and 200 mM NaCl, with 4, 6, 8, or 10 wt% PGPR. D-(30–80) refers to W/O emulsions containing 8 wt% PGPR and 200 mM NaCl, with 30, 50, or 80% (*v*/*v*) aqueous phase. Na-(10–300) refers to W/O HIPEs containing 8 wt% PGPR and 80% (*v*/*v*) aqueous phase, with 10, 50, 100, 200, 300 mM NaCl.

Sample	*ln*(*k*) = −*Ea/RT* + *ln*(*A*)	R^2^	*E_a_* (KJ/mol)	*A*
P-4	y = −4730.15402x + 15.75783	0.98	39.33	7.02 × 10^6^
P-6	y = −4628.61652x + 15.25867	0.99	38.48	4.23 × 10^6^
P-8	y = −4810.96413x + 15.69495	0.99	40.00	6.58 × 10^6^
P-10	y = −5407.27597x + 17.47865	0.98	44.96	3.93 × 10^7^
D-30	y = −5716.4043x + 18.20629	0.99	47.55	8.89 × 10^7^
D-50	y = −5744.46278x + 18.41839	0.98	47.77	1.01 × 10^8^
D-80	y = −4810.96413x + 15.69495	0.99	40.00	6.58 × 10^6^
Na-10	y = −4870.84828x + 15.99132	0.99	40.49	8.80 × 10^6^
Na-50	y = −4538.96615x + 14.61999	0.99	37.75	2.23 × 10^6^
Na-100	y = −4488.16459x + 14.47879	0.99	37.31	1.95 × 10^6^
Na-200	y = −4810.96413x + 15.69495	0.99	40.00	6.58 × 10^6^
Na-300	y = −5055.40355x + 16.56701	0.99	42.03	1.56 × 10^7^

## Data Availability

The original contributions presented in the study are included in the article; further inquiries can be directed to the corresponding author.
